# Dulaglutide (Trulicity)-Induced Acute Pancreatitis: A Case Report

**DOI:** 10.7759/cureus.38630

**Published:** 2023-05-06

**Authors:** Abu baker Khan, Aimal Shah, Saad Ahmad, Moiz I Khan, Ahsan Amir

**Affiliations:** 1 Internal Medicine, Ayub Teaching Hospital, Abbottabad, PAK; 2 Medicine, Nazareth Hospital - Trinity Health Mid-Atlantic, Philadelphia, USA; 3 Internal Medicine, Nazareth Hospital, Philadelphia, USA; 4 Orthopedic Surgery, Taj Medical Center, Nowshera, PAK; 5 Accident and Emergency, Medical Teaching Institution (MTI) Divisional Headquarter (DHQ) Teaching Hospital, Dera Ismail Khan, PAK; 6 Internal Medicine, Khyber Medical College, Peshawar, PAK

**Keywords:** dose-dependant, weight loss drug, diabetes, acute pancreatitis, trulicity

## Abstract

The article discusses the use of dulaglutide (Trulicity) in treating type 2 diabetes mellitus. Dulaglutide is a synthetic analog of glucagon-like peptide (GLP-1) that binds to GLP-1 receptors, enhancing insulin secretion and reducing postprandial glucagon and food intake. Dulaglutide has a longer half-life than GLP-1, making it more clinically useful. The recommended dosage of dulaglutide is 0.75 mg/0.5 mL subcutaneously once weekly, which can be increased as needed for adequate glycemic control. We describe a case of acute pancreatitis in a 37-year-old male with a past medical history of type 2 diabetes mellitus who was admitted for epigastric pain radiating to the back. Lipase level was elevated at 1508, and a computed tomography (CT) scan of the abdomen showed fat stranding around the pancreas consistent with pancreatitis. The patient was on dulaglutide (Trulicity) at 0.75 mg q. weekly for about two years; this dose was increased to 1.5 mg q. weekly two months ago. He developed symptoms of abdominal pain, nausea, and vomiting after receiving the last dose of Trulicity, which was two weeks before he presented to the emergency department as a cause of acute pancreatitis. Dulaglutide use has been known to cause a mild elevation of pancreatic enzyme levels; there have been few reported cases of dulaglutide-associated acute pancreatitis in the literature. The case report highlights the adverse effects of dulaglutide in diabetic patients and the importance of monitoring pancreatic enzyme levels in patients taking dulaglutide.

## Introduction

Dulaglutide (Trulicity) is an analog of glucagon-like peptide (GLP-1) that binds to GLP-1 receptors, enhancing the glucose-dependent secretion of insulin. It also slows gastric emptying, causing early satiety and reducing postprandial glucagon and food intake [1]. GLP-1 is normally produced in the body by the L cells of the small intestine and colon, with GLP-1 receptors expressed in various tissues, including beta-pancreatic cells, pancreatic ducts, gastric mucosa, lungs, heart, skin, and hypothalamus. GLP-1 exerts its effects by stimulating the glucose-dependent release of insulin from pancreatic islet cells [2]. The half-life of GLP-1 is approximately one to two minutes, and it is degraded by the enzyme dipeptidyl peptidase 4 (DPP-4). Synthetic GLP-1 analogs like Trulicity are designed to be resistant to degradation by DPP-4 and have a longer half-life, which facilitates their clinical use [3]. Dulaglutide is typically used in combination with other oral hypoglycemic medications, such as metformin, and has been found to cause weight loss due to its ability to cause early satiety by slowing gastric emptying. This weight loss potential has made GLP-1 analogs widely used in diabetic patients [4].

The recommended dosage of Trulicity (dulaglutide) is 0.75 mg/0.5 mL subcutaneously once weekly, and the dose can be increased to 1.5 mg once weekly after four to eight weeks to achieve adequate glycemic control. A maximum dose of 4.5 mg once weekly can be administered. The digestive system is commonly affected by the adverse effects of dulaglutide, resulting in symptoms such as nausea, vomiting, abdominal discomfort, and diarrhea. Additionally, dulaglutide usage has been linked to increased levels of amylase and lipase, but these levels typically remain within normal ranges. However, there is a rare but serious adverse effect of dulaglutide use, which is pancreatitis [5].

We report an interesting case of dulaglutide-induced acute pancreatitis in a patient with diabetes.

## Case presentation

**Figure 1:**
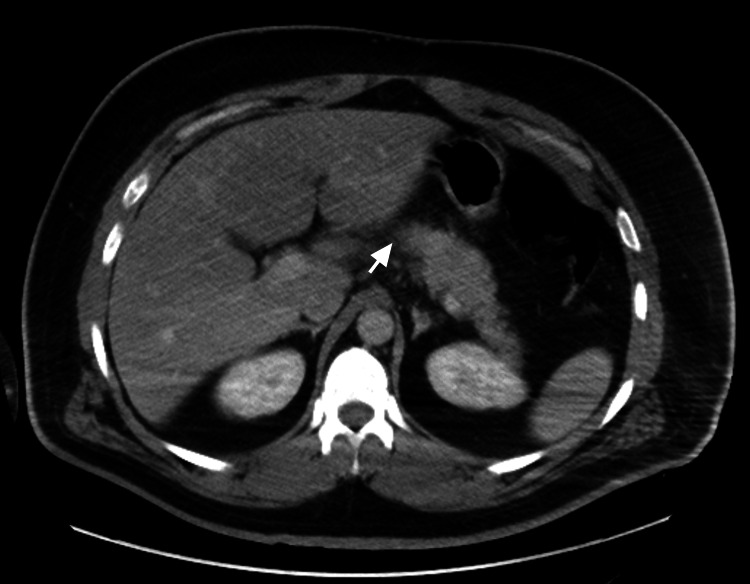
Computed tomography scan without contrast of abdomen. CT scan of the abdomen without contrast showing peripancreatic fat stranding (white arrow), findings consistent with acute pancreatitis.

A 37-year-old male with a past medical history of insulin-dependent diabetes mellitus and congenital clubfoot and a past history of multiple surgeries presented to the emergency room (ER) with a chief complaint of abdominal pain associated with nausea and vomiting. The patient's abdominal pain started a few days ago in the epigastric region and now radiates to the back. It was dull in character and constant in nature, and eating and drinking were exacerbating the pain. Over-the-counter painkillers did not relieve his pain. The patient has no fever and has normal bowel movements. In the ER labs, lipase was elevated to 1508 U/L. A computed tomography (CT) scan of the abdomen showed fat stranding around the pancreas, consistent with acute pancreatitis (Figure 1). He was admitted to the hospital for the management of acute pancreatitis.

Abdominal ultrasound of the right upper quadrant showed no intrahepatic duct dilation and no cystic or solid hepatic mass. The common bile duct measured 0.2 cm, and no common duct stones were present. The gallbladder was distended, and there was no evidence of acute cholecystitis. The serum alcohol level was found to be normal at less than 3, and the triglyceride levels were normal at 69 mg/dl. The patient is a nonsmoker, does not use illicit drugs, occasionally takes alcohol, and has an active lifestyle.

On reviewing the patient's home medication, he was taking atorvastatin 20 mg every afternoon, pantoprazole 40 mg orally, insulin glargine 10 units subcutaneously daily, insulin Lispro 20 units subcutaneously before breakfast, and dulaglutide 1.5 mg subcutaneously weekly. On further interviewing, he stated that his dose was increased two weeks prior from 0.75 mg/week to 1.5 mg/week. He developed severe nausea and vomiting after he received the medication. The symptoms of nausea and vomiting resolved on their own, but subsequently, after a few days, he developed abdominal pain.

On the floor, the patient's diabetes was controlled with insulin glargine and lispro; dulaglutide was discontinued, and the patient's lipase was trended. He received symptomatic management with IV fluids and as-needed pain medication. His lipase trended upward (Table 1).

**Table 1:** Trending lipase after discontinuing Trulicity

Day	Lipase	Units/liter (U/L)
Day 1	1508	U/L
Day 2	998	U/L
Day 3	868	U/L
Day 5	439	U/L

He continued to have upper abdominal pain, and an upper endoscopy was done, which was negative for any acute pathology like gastritis, peptic ulcer disease, etc. There was a significant improvement in pain, so he was discharged after a few days with an indication of discontinuing dulaglutide, as this therapy would be the cause of the acute pancreatitis condition.

## Discussion

Trulicity (dulaglutide) is an oral hypoglycemic medication used to treat type 2 diabetes [3]. It acts on the pancreas, stimulating beta pancreatic cells and causing the release of insulin. In Global AWARD phase 3 studies, dulaglutide has shown superior or non-inferior management of blood sugar levels in a vast population of people with type 2 diabetes at different stages of the disease, as compared to various active comparator drugs like metformin, sitagliptin, exenatide, liraglutide, and insulin glargine [6]. Different studies have shown weight loss with this medication, making it a popular choice for the treatment of type 2 diabetes. One of the documented side effects of Trulicity is acute pancreatitis. Our patient had been receiving Trulicity 0.75 mg/week subcutaneously for two years without any symptoms; however, after increasing the dose to 1.5 mg/week subcutaneously, he developed acute pancreatitis.

Dulaglutide can also cause a mild elevation in serum amylase and lipase levels, which is not considered significant and is therefore rarely reported. Full-blown dulaglutide-induced acute pancreatitis is not common, and only a few cases have been reported in the literature [7,8]. On the other hand, exenatide, another GLP-1 agonist used to treat type 2 diabetes, has been found to have a six-fold higher chance of causing pancreatitis based on reported cases [9]. The exact pathophysiologic mechanism of pancreatic enzyme elevation with GLP-1 agonists is not known, but it is hypothesized that continuous activation of the GLP-1 receptor due to an increased level of external or internal ligands amplifies the secretion of digestive enzymes from acinar cells. This interplay could involve the parasympathetic component of the autonomic nervous system by managing the secretion of the exocrine pancreas, which can be influenced by incretins through GLP-1 receptors expressed in certain areas of the system [10].

During the AWARD-2 clinical trial, three instances of pancreatitis were confirmed in patients who were treated with dulaglutide, with one patient receiving a 0.75 mg dose and the other two receiving a 1.5 mg dose. One of the acute cases was observed in an asymptomatic patient who received the 0.75 mg dose of dulaglutide, with the diagnosis being made a day after treatment due to abnormal laboratory results prior to treatment [11]. Clinical trials of dulaglutide have reported pancreatitis as a possible adverse reaction, with a rate of 1.4 cases per 1,000 patient-years in dulaglutide-exposed individuals compared to 0.88 cases per 1,000 patient-years in non-incretin control groups [12].

Nauck et al. conducted a cohort study with a sample size of 6005 to assess the risk of acute pancreatitis in diabetic patients receiving the GLP-1 agonist dulaglutide, placebo, and active comparators. The impact of dulaglutide on pancreatic enzymes was more significant and had a dose-dependent effect. For lipase, the median changes were between 3.0 and 6.0 units/L for the 0.75 mg dosage and 5.0 to 7.0 units/L for the 1.5 mg dosage, while the changes for p-amylase were slightly lower. This increase in pancreatic enzyme levels associated with dulaglutide resulted in more patients having values ≥2× upper limit of normal (ULN) and ≥3× ULN for both enzymes compared to placebo. However, after the treatment was discontinued, the pancreatic enzyme levels returned to almost baseline levels within four weeks. Out of 4006 patients treated with dulaglutide, three patients reported signs and symptoms of acute pancreatitis, and all three of them had pre-existing increased levels of pancreatic enzymes. It could not be concluded whether the observed acute pancreatitis was due to dulaglutide's adverse effect or any other pre-existing abnormality [13]. Similarly, Li et al. conducted a systematic review and meta-analysis of 60 studies on treatment with GLP-1 receptor agonists or dipeptidyl peptidase-4 (DPP-4) inhibitors and found no available evidence to show that these drugs can increase the risk of pancreatitis [14]. Despite the current body of evidence, it is still inconclusive, and thus more carefully designed and conducted observational studies are needed to determine the scope of the issue definitively. A study is being conducted in Europe to evaluate the vascular and pancreatic safety of various diabetes drugs, such as thiazolidinediones (TZDs), incretins, and amylin analogs, in individuals with type 2 diabetes. The study utilizes surveillance and observational methods, and its results are expected to offer more conclusive evidence on the matter [12].

## Conclusions

Given that the patient developed acute pancreatitis after his dose of dulaglutide was increased, this suggests that there is a dose-dependent relationship between dulaglutide and acute pancreatitis, but it is unclear if decreasing the dose would prevent this. Although the use of dulaglutide has been associated with a slight increase in pancreatic enzyme levels, there is limited evidence in the literature regarding cases of acute pancreatitis associated with dulaglutide. Our case report highlights one such adverse effect of dulaglutide in a patient. For now, we should pay close attention to the side effect profile of dulaglutide use and, at the same time, not hesitate to use it in diabetic patients as it has proven beneficial effects in controlling diabetes. To obtain significant findings, it is essential to gather data from several prospective studies, as pancreatitis has a very low incidence in the general population.
